# Cost-effectiveness of teduglutide in adult patients with short bowel syndrome – a European socioeconomic perspective

**DOI:** 10.1016/j.ajcnut.2024.02.031

**Published:** 2024-03-01

**Authors:** Evelyn Walter, Christopher Dawoud, Elisabeth Hütterer, Anton Stift, Felix Harpain

**Affiliations:** 1 IPF Institute for Pharmaeconomic Research, Vienna, Austria; 2 Division of Visceral Surgery, Department of General Surgery, Medical University of Vienna, Vienna, Austria; 3 Division of Oncology, Department of Internal Medicine I, Medical University of Vienna, Vienna, Austria

**Keywords:** cost-effectiveness, socioeconomic, GLP-2, teduglutide, short bowel syndrome

## Abstract

**Background:**

Short bowel syndrome with intestinal failure (SBS-IF) is a rare but devastating medical condition. An absolute loss of bowel length forces the patients into parenteral support dependency and a variety of medical sequelae, resulting in increased morbidity and mortality. Interdisciplinary treatment may include therapy with the effective but expensive intestinotrophic peptide teduglutide.

**Objectives:**

A time-discrete Markov model was developed to simulate the treatment effect [lifetime costs, quality-adjusted life years (QALYs), and life years (LYs)] of teduglutide plus best supportive care compared with best supportive care alone in patients with SBS-IF.

**Methods:**

The health status of the model was structured around the number of days on PS. Clinical data from 3 data sets were used: *1*) an Austrian observational study (base case), *2*) pooled observational cohort studies, and *3*) a prospective study of teduglutide effectiveness in parenteral nutrition-dependent short bowel syndrome subjects. Direct and indirect costs were derived from published sources. QALYs, LYs, and costs were discounted (3% per annum).

**Results:**

Under the base case assumption, teduglutide is associated with costs of 2,296,311 € per patient and 10.78 QALYs (13.74 LYs) over a lifetime horizon. No teduglutide is associated with 1,236,816 € and 2.24 QALYs (8.57 LYs). The incremental cost-utility ratio (ICUR) amounts to 123,945 €. In case of the pooled clinical data set, the ICUR increases to 184,961 €. If clinical data based on the study of teduglutide effectiveness in parenteral nutrition-dependent short bowel syndrome subjects were used, the ICUR increased to 235,612 €.

**Conclusions:**

Teduglutide in treating patients with SBS-IF meets the traditional cost-effectiveness criteria from a European societal perspective. Nevertheless, the varying concentrations of teduglutide efficacy leave a degree of uncertainty in the calculations.

## Introduction

Short bowel syndrome (SBS) is a heterogenic medical condition where patients suffer from impaired intestinal absorption because of absolute loss of bowel as a result of surgical resection or disease-associated destruction of the bowel. patients with SBS with intestinal failure (IF) denote a decreased quality of life (QoL) and an increased morbidity and mortality [caused by potentially life-threatening complications such as central line–associated bloodstream infections (CLABSIs), central venous thrombosis and IF-associated liver disease] because of their dependency of parenteral support (PS), consisting of parenteral nutrition (PN) and/or fluid and micronutrient support [1]. Despite a rather precise definition of the disease, the true prevalence and incidence of SBS in adults are difficult to determine as there is a lack of consistently applied disease criteria, the nonexistence of reliable databases, and the fact that estimations highly vary by region and mostly refers to patients receiving long-term PS [2]. Nevertheless, most studies classify SBS-IF as an ultraorphan disease with prevalence rates far below the internationally accepted threshold of 20 per 1,000,000 population [[3], [4], [5], [6]]. The interindividual heterogeneity of clinical presentation and the varying extent of PS dependency may be explained by the nutritive-metabolic deficit caused by differences in the remnant anatomy of the intestine [6]. Interdisciplinary intestinal rehabilitation programs focus with their therapy regimens on the optimization of the remnant intestinal function [7]. Nevertheless, nearly half of the patients remain dependent on PS [8]. Teduglutide is a degradation-resistant glucagon-like peptide 2 analogue that enhances the functional and structural capacity of the intestine [9]. Clinical studies were able to demonstrate that patients with SBS-IF who were treated with teduglutide were able to reduce and even discontinue PS (enteral autonomy) with varying levels of success [6,10]. Unfortunately, teduglutide is expensive, with an estimated cost of over 237,680 € per patient per year (Austrian classified index of goods I 2022). On the contrary, SBS-IF and its secondary medical sequelae have their high costs (e.g., PS dependency with ∼13,000–71,000 €/y [11,12], CLABSI event mounting ≤∼25,000 €/event [13]). Evaluating the cost-effectiveness of ultraorphan drugs needs to balance disease severity and uncertainty. European countries such as the United Kingdom, Netherlands, Norway, and Sweden give extra consideration to severe diseases by either adopting shortfall methods or implicitly considering higher thresholds for more severe diseases. The National Institute of Health and Care Excellence (NICE) appraises treatments for rare, severely disabling diseases with a high unmet need, using a significantly higher willingness to pay (WTP) thresholds equivalent to £100,000–300,000 per quality-adjusted life year (QALY) gained [14]. Raghu et al. [15] studied the cost-effectiveness of teduglutide in the United States health care system and compared standard of care SBS-IF management with and without teduglutide with a Markov model. In an adult as well as a pediatric setting teduglutide failed to meet a traditional cost-effectiveness threshold [16]. Nonetheless, they compared only direct health care costs and thereby disregarded the beneficial socioeconomic effect of the potential reintegration into the labor market of patients with SBS-IF reaching enteral autonomy. Furthermore, PS weaning probability was only interpolated from the original pivotal randomized controlled study of teduglutide effectiveness in PN-dependent short bowel syndrome subjects (STEPS) by Jeppesen et al. [17]. However, recent trials on PS weaning under teduglutide therapy reveal high variation in clinical response rates, with some studies reporting much higher rates of PS weaning and enteral autonomy [10,[18], [19], [20], [21]]. This study aimed for an evaluation of the cost-effectiveness of teduglutide therapy in a setting of European adult patient with SBS-IF considering different teduglutide response rate scenarios from a societal perspective in order to evaluate the socioeconomic impact associated with the treatment of patients with SBS-IF with teduglutide.

## Methods

### Model design

**FIGURE 1:**
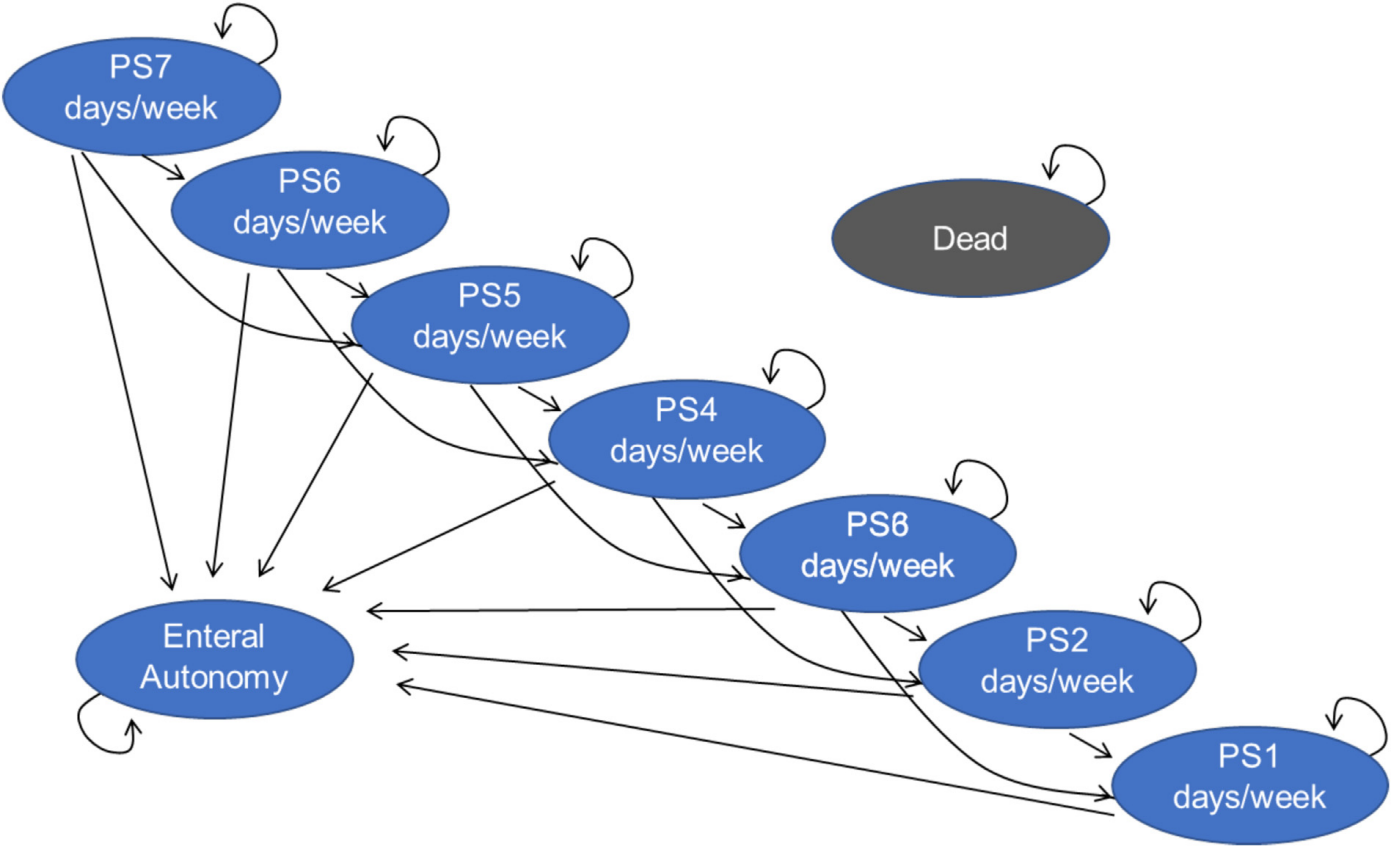
Markov model. Markov process with a cycle length of 4 wk and 9 defined states. PS, parenteral support.

We used a Markov simulation model based on published models [15,22] to evaluate the costs (€) and effectiveness (QALY) of the treatment of patients with SBS-IF with teduglutide plus best supportive care (BSC) compared with BSC alone. The health states of the model were structured around the number of days on PS. The model incorporates 8 core PS states, from a requirement for PS 7 d/wk (PS7) through no requirement for PS (PS0). A 28-d Markov cycle was chosen to be in keeping with the STEPS trial monthly assessment schedule. All patients analyzed in the model were distributed according to the health states PS3–PS7. During each model cycle, patients were either weaned by 1 or 2 d/wk or remained in the same PS states. A patient could also die from any cause from any PS state. The model allowed patients to discontinue therapy with teduglutide at any time; then PS transitions developed according to the BSC alone arm. Adverse events (AEs) were considered in both treatment arms. See Figure 1 for the model structure.

In order to delineate the effect of teduglutide based on different treatment approaches and study settings, the following transition probabilities from retrospective observational studies, as well as prospective randomized controlled clinical trials, were generated and incorporated into 3 different scenarios on the basis of the following sources (each treatment scenario had the same model structure but differed in the transition probabilities with regard to response to teduglutide therapy):

1) The observational study by Harpain et al. [10] to picture the Austrian clinical practice (base case).
2) Pooled transition probabilities of 6 systematically selected observational studies (Supplemental Table 1 and Supplemental Figure 1) [10,[18], [19], [20], [21],^23^].
3) The STEPS randomized controlled Phase III trial and its open-label extension (STEPS II) [17,24].

For this purpose, a systematic review was carried out according to Cochrane Collaboration guidelines (www.cochrane.org). The search strategy was carried out using the MEDLINE database and the Cochrane Central Register of Controlled Trials. Studies were included in this review if they met the following criteria: study population of patients with SBS-IF 18 y of age or older; study design of either randomized controlled trial or observational cohort studies; ≥10 patients treated with teduglutide. Two independent reviewers (EW and FH) screened titles and abstracts; articles that did not fulfill the inclusion criteria were excluded.

A societal perspective was chosen for the analysis because relevant consequences also appear outside the health care system. Therefore, direct and indirect costs were considered in the model. The primary effect when using teduglutide was the increased likelihood of reduced PS per week.

The costs were calculated for each of the cycles in Euros (€) for the year 2022. Direct and indirect costs were derived from published sources (TABLE 1, TABLE 2). The model provided cumulative costs, outcomes expressed as QALYs and life years (LYs) within a lifetime horizon, as well as the incremental cost-utility ratio (ICUR) and incremental cost-effectiveness ratio (ICER), which can be interpreted as additional costs per QALY or LY gained. QALYs, LYs, and costs were discounted (3% per annum).

**TABLE 1:** Model inputs: direct cost

Medication			
Product name	Active substance	Per unit €	Source
Revestive (5 mg in 0.5 mL) 28 vials	Teduglutide	18,233.05	Austrian classified index of goods I 2022 (Austrian positive list)
PS costs	Units	Per unit €	
SmofKabiven Low Osmo peripheral 850 mL	5	323.55	Austrian classified index of goods I 2022
Smofkabiven Ntense1012 mL	4	257.55	
Smofkabiven Ntense 1518 mL	4	331.10	
SmofKabiven 493 mL	6	248.65	
SmofKabiven 986 mL	4	200.00	
SmofKabiven 1477 mL	4	283.00	
SmofKabiven 1970 mL	4	354.15	
Nutriflex Lipid peri 1250 mL	5	227.25	
Nutriflex Lipid peri 1875 mL	5	265.40	
	5	314.45	
Ringer - lactate “Fresenius” - solution for infusion 500 mL	10	9.60	
Supplementation costs	Units	Per unit €	
Soluvit	10	11.38	Austrian classified index of goods I 2022
Vitalipid	10	10.20	
Tracel	5	14.88	
Dipeptiven 100 mL	10	38.04	
Omegaven 100mL	10	18.71	
Magnesium gluconicum G.L. 1000 mg	5	7.65	
Monitoring costs	Frequency per cycle	Cost per intervention €	
Outpatient physician	1	21.62	Tariff catalogs of the 9 ÖGKs (weighted population average)
Blood count	1	9.21	
Comprehensive metabolic profile	1	20.66	
Triglyceride concentration	1	3.30	
Pantoprazole	1	4.80	Austrian classified index of goods I 2022
Kreon 25000 IE (50 tablets)	28 tablets	21.70	
Enterobene 2 mg (50 tablets)	28 tablets	8.30	
Quantalan 4 g (50 tablets)	28 tablets	29.55	
Novalgin (100 tablets)	28 tablets	12.05	
Nephrotrans 840 mg (100 tablets)	28 tablets	21.00	
Home nursing per hour	1	82.50	Average fee [34]
Adverse event costs	Cost per event €		
Abdominal pain	616.68		Tariff catalogs of the 9 ÖGKs (weighted population average), LKF Modell 2022 (Austrian DRG-System), Austrian classified index of goods I 2022
Nausea	614.37		
Gastrointestinal stoma change	3408		
Abdominal distension	975		
Peripheral edema	747		
Urinary tract infection	1,256.18		
Flatulence	0		
Vomiting	614.72		
Fatigue	1,219.82		
Pyrexia	1806		
Diarrhea	593.89		
Weight increase	952		
Dyspnea	696		
Nasopharyngitis	1697		
Central line–associated bloodstream infection	25,436.80		Blot et al. [13] 2005

Abbreviations: PS, parenteral support; ÖGK, Österreichische Gesundheitskasse; LKF, leistungsorientierte Krankenanstaltenfinanzierung; DRG, diagnosis related groups.

**TABLE 2:** Model inputs: indirect cost

Return to work		
Health state	Return to work per year	Source
PS7 d/wk	16.43%	Calculated from [31]
PS6 d/wk	28.37%	
PS5 d/wk	40.31%	
PS4 d/wk	52.25%	
PS3 d/wk	64.19%	
PS2 d/wk	76.12%	
PS1 d/wk	88.06%	
PS0 d/wk (independent of PS)	100.00%	
<5 times/wk vs. 5–6 times/wk OR	2.26 (1.02–5.02)	Samuel et al. [31] 2019
Returning to employment	34%	Samuel et al. [31] 2019
Formular: (X _PN < 5d_ ∗ OR) + X _PN ≥ 5d_ = 0.34		
Gross salary per year	35,272.00 €	Statistik Austria [32] 2021
Caregiver time (professional caregiver)		
Patients with caregivers in %	63%	Beurskens-Meijerink et al. [33] 2020
Home nursing in h/d	4	Raghu et al. [15] 2020
Caregiver salary per h	7.64 €	Statistik Austria [32] 2021
Caregiver work absenteeism (family member)		
Employed caregiver	60%	Jeppesen et al. [35] 2022
Absenteeism from work	7%	

Abbreviations: OR, odds ratio; PN, parenteral nutrition; PS, parenteral support.

To assess the robustness of our findings, we carried out deterministic and probabilistic sensitivity analyses.

The model was constructed and analyzed using Microsoft Excel 365 version 16.77.1. The analysis was conducted in consideration of the Modeling Good Research Practices published by the International Society for Pharmacoeconomics and Outcomes Research Task Force [25] and the Austrian health economic guidelines [26].

### Study population

The study population met the inclusion criteria of the STEPS study: patients with SBS-IF who were stable following a period of intestinal adaptation after surgery. Patients were adults [mean age (range): 50.3 (18–82)] after intestinal resection, mainly because of vascular disease and Crohn disease. Fifty-four percent of patients had female sex. The mean BMI (in kg/m^2^) was 22.4 (range: 17.6–29.8). The mean time receiving PS was 6.3 y, with a mean parenteral volume of 1887 mL/d on average 5.9 PS d [17]. With bootstrapping statistics, Monte Carlo simulations of 1000 hypothetical patients were carried out on the basis of all input variables (see chapter “sensitivity analysis”).

### Clinical effects and transition probabilities

Transition probabilities were obtained from published data and are listed in Table 3. In the model, the starting cohort has a minimum level of PS dependency of ≥3 d/wk, reflecting the inclusion criteria of the STEPS trial. The distribution of PS days in the first cycle corresponded to the baseline distribution of the average number of days on PS in the STEPS trial [22]. The model used different weekly PS weaning probabilities up to week 24 and between week 24 and 2.5 y. After that, no improvement was assumed, and the distribution remained unchanged.

**TABLE 3:** Clinical input data

Proportion of adult patients by PS distribution at baseline			
	Teduglutide %	No teduglutide %	Source
PS7 d/wk	52	52	Scotland et al. [22] 2017
PS6 d/wk	18	18	
PS5 d/wk	6	6	
PS4 d/wk	13	13	
PS3 d/wk	11	11	
PS2 d/wk	0	0	
PS1 d/wk	0	0	
PS0 d/wk	0	0	
Probabilities of weaning up to week 24			
Harpain et al. 2022 By 1 d of PS in 24 wk	14.29		Harpain et al. [10] 2022
By 2+ d of PS in 24 wk	8.79		
Without improvement	7.69		
PS independence	69.23		
Pooled			
By 1 d of PS in 24 wk	31.76		Harpain et al. [10] 2022, Puello et al. [19] 2020, Joly et al. [20] 2019, Pevny et al. [21] 2018, Schoeler et al. [23] 2018, Lam et al. [18] 2018
By 2+ d of PS in 24 wk	19.54		
Without improvement	23.43		
PS independence	25.27		
STEPS 1			
By 1 d of PS in 24 wk	33.33	15.38	Jeppesen et al. [17] 2012, Jeppesen et al. [38] 2011, Raghu et al. [15] 2020.
By 2+ d of PS in 24 wk	20.51	7.69	
Without improvement	46.15	76.92	
PS independence	0		
Transition probabilities per cycle of weaning from week 24 to 2.5 y			
By 1 d of PS in 24 wk	0.03		Harpain et al. [10] 2022, splitting analogous to STEPS
By 2+ d of PS in 24 wk	0.23		
PS independence	10.14		
Without improvement	89.61		
Pooled			
By 1 d of PS in 24 wk	0.21		Harpain et al. [10] 2022, Puello et al. [19] 2020, Joly et al. [20] 2019, Pevny et al. [21] 2018, Schoeler et al. [23] 2018, Lam et al. [18] 2018, splitting analogous to STEPS
By 2+ d of PS in 24 wk	2.39		
PS independence	1.93		
Without improvement	95.47		
STEPS 2			
By 1 d of PS in 24 wk	0.06		Seidner et al. [30] 2020
By 2+ d of PS in 24 wk	0.61		
PS independence	1.28		
without improvement	98.04		
Proportion of patients who discontinue teduglutide because of insufficient response			
	Week 24 (%)	Month 12/24 (%)	
PS7 d/wk	27.0	23.0/17.0	Scotland et al. [22] 2017
PS6 d/wk	17.0	0.0	
PS5 d/wk	33.0	0.0	
PS4 d/wk	0.0	0.0	
PS3 d/wk	67.0	0.0	
PS2-PS0 d/wk	0.0	0.0	
Adverse events reported in >5%			
	Teduglutide %	No teduglutide %	
Abdominal pain	30.95	23.26	Jeppesen et al. [17] 2012
Nausea	28.57	18.60	
Gastrointestinal stoma change	23.81	6.98	
Abdominal distension	21.43	2.33	
Peripheral edema	16.67	4.65	
Urinary tract infection	14.29	9.30	
Flatulence	11.90	6.98	
Vomiting	11.90	9.30	
Fatigue	9.52	6.98	
Pyrexia	9.52	9.30	
Diarrhea	7.14	11.63	
Weight increase	7.14	6.98	
Dyspnea	7.14	0.00	
Nasopharyngitis	7.14	0.00	
Central line–associated bloodstream infection PS7 d/wk PS6 d/wk PS5 d/ wk PS4 d/wk PS3 d/wk PS2 d/wk PS1 d/wk	18.1 16.8 15.5 14.2 12.9 11.6 10.3		Calculated based on STEPS data. 16.67% in the teduglutide arm (5.9 PS days) and 16.28% in the control arm (5.6 PS days) β1 = –0.013

Abbreviations: PS, parenteral support; STEPS, study of teduglutide effectiveness in parenteral nutrition-dependent short bowel syndrome subjects.

#### Up to week 24

The weekly PS weaning probability within the first 24 wk was interpolated from the following selected data sources: *1*) The observational study by Harpain et al. [10] documented the PS reduction at week 24. Transition probabilities were determined for the 4-wk transitions. *2*) In the case of the pooled data, either results for week 24 were published or a time adjustment was necessary, which was done with the following formula:

$$=1-\exp\;(-1*\left(-\frac{\ln\left(endpoint\right)}{median\;study\;follow-up/24}\right)$$

*3*) Data from the 24-wk STEPS trial were used. Weekly PS weaning probabilities without teduglutide were derived from the STEPS study’s control group [17].

#### Week 24–2.5 y

A similar approach was used for the time between 24 wk and 2.5 y: *1*) 4-wk transition probabilities were calculated from the observational data with a median follow-up of 107 wk by Harpain et al. [10]. *2*) From the pooled observational cohort studies, data are available for a median follow-up of between 24 and 107 wk; from this, 4-wk transitional probabilities were derived, which are applied over the period between week 24 and 2.5 y. *3*) Finally, transitions for patients who continued teduglutide and followed in the open-label extension to STEPS (STEPS 2) were incorporated. This described set of transition probabilities was applied for patients on treatment in the teduglutide arm up to week 128 or cycle 32. Patients who did not receive teduglutide remained in their current health states after week 24 (cycle 6), as no further observations on patients assigned to placebo beyond 24 wk were available.

The model considered the discontinuation of teduglutide therapy. Discontinuation rates for week 24, month 12, and month 24 were taken from the Aberdeen Health Technology Assessment. Group publication and correspond to the NICE company submission for Revestive [22,27].

A time adjustment to the Markov cycle length was performed for all the presented probabilities.

### AE

AE experienced by ≥5% of patients was included in our model according to the STEPS trial [17]. CLABSIs were only attributed to those patients who receive PS. In accordance with the literature, it was assumed that the probability of CLABSIs decreases with a reduction in PS days [28].

### Survival

Because of the limited data available from the STEPS trial, survival was modeled using the published observational data of 268 patients with nonmalignant SBS. Amiot et al. [29] report a large series of adult patients with SBS over a 25-y period of follow-up to better define the long-term survival of patients with SBS on PS. Patient characteristics compared with the STEPS trial were similar. The observational study includes all courses of mortality, with 13% attributable to PS-related reasons, which means that the mortality rate is only partly because of central line removal. Results demonstrated that the 10-y survival was significantly higher in patients who became PS-free compared with those who remained PS-dependent (67.0% ± 0.6% compared with 40.7% ± 0.5%, *P* < 0.001) [29]. Based on these findings, mortality was modeled under consideration of PS dependency, which means any requirement (1–7 d) compared with no requirement (0 d), using parametric survival curves fitted to the Kaplan-Meier survival analysis. The curve was created based on the Markov cycle length between 100% alive in the first cycle and 67% alive after 10 y (last cycle). Therefore, an extrapolation of the mortality curves over the remaining lifetime was required. To extrapolate the Kaplan-Meier survival curves to a lifetime horizon, the Weibull curve was applied based on the results of the model fitting statistics (i.e., Akaike information criterion, Bayesian information criterion) (Supplemental Figure 2).

### Cost assessment

The cost assessment was based on the assignment of costs to the health states. The costs of each health state were determined by the resource utilization associated with a health state. In order to estimate the costs of both comparators in Austria, direct medical costs and indirect costs were included in the analysis.

#### Direct costs

Direct costs comprise teduglutide costs, PS costs plus supplementation, monitoring costs, and AE costs (Table 1). Teduglutide (Revestive) costs were evaluated based on the reimbursement price and extracted from the official Austrian classified index of goods (“Warenverzeichnis Apothekerverlag”) of 9/2022 (Austrian classified index of goods I 2022). PS volume subgroups (≤9 L/wk, >9–18 L/wk, and >18 L/wk) were applied to calculate average PS costs per week. The average administered volume from the STEPS study was used as a basis for calculation for the first 24 wk. This resulted in an average of 12.01 L/wk. From week 24 onward, volumes of the STEPS 2 study were utilized, which resulted in an average volume of 12.20 L/wk [30]. The average PN cost per liter was calculated using products administered in everyday clinical practice. This resulted in an average cost of 47 €/L (Austrian classified index of goods I 2022). In addition, patients with PN receive supplementation, which includes the following components: Soluvit or Vitalipid 2 units per PS day, Tracel, Dipeptiven, Omegaven, and magnesium 1 unit per PS day. This resulted in an average cost of 84 €/d for supplementation (Austrian classified index of goods I 2022).

In addition, the monitoring costs were considered. Treatment-specific resource use data were obtained from relevant product information and the literature and are composed as follows: *1*) 1 outpatient physician visit per month, *2*) laboratory tests (blood count, comprehensive metabolic profile, and triglyceride concentration), *3*) medication costs, *4*) home nursing when receiving PS 1 time/mo [15].

The percentage of patients exhibiting an AE is displayed in Table 3. Average costs per complication were calculated according to the expected setting of care as an average value.

#### Indirect costs

Because the average age of the study population was 50.3 (range: 18–82) y, the majority was of working age. For this reason, it was considered relevant to include indirect costs in the analysis. Indirect costs comprised work absenteeism, caregiver costs, and work absenteeism costs of family members caring for patients. Although there is a growing body of evidence describing the disease-associated burden of patients on PS, only a few surveys examine the ability to maintain employment while receiving PS. Samuel et al. [31] analyzed the effect of PS on employment and factors associated with the likelihood of maintaining or returning to employment while receiving PS. Results revealed that before their illness and commencing PS, 94% of patients were in full or part-time employment [32]. Because this percentage is higher than the Austrian employment rate of 85.2%, we used national figures as the maximum value [32]. At the time of starting PS, 36% were in employment and it was observed that 34% of patients were able to return to employment in the observed time period. Furthermore, for PS of <5 d/wk and 5–6 d/wk, an odds ratio of 2.26 associated with return to work after the commencement of PS is stated [31]. The probabilities of returning to work as a function of PS days are shown in Table 2. The lost productivity is assessed based on the average median income of the age groups 40–59, as no re-entry was possible after that.

Caregiver costs were considered as a further indirect cost component. A cross-sectional study analyzing the caregiver burden in PS-dependent patients showed that 63% of all patients require a caregiver [33]. Home nursing time of caregivers was estimated to be 4 h/d while receiving PS, with the cost estimated by the hourly wage calculated based on the average median income [15]. The daily rate for caregivers was between 65 € and 100 €, depending on the amount of care required and the training of the care staff. This daily rate is determined after an initial anamnesis interview and can be adjusted upwards if there is an increased need for care. Therefore, we have decided to use an average value of 83 € (the data were derived from an organization 24 h care Vienna and Lower Austria) [34].

Work absenteeism costs of family members caring for patients with PS were also considered as an indirect cost component. A European cross-sectional survey using the Workers Productivity and Activity Impairment Questionnaire: Specific Health Problem has assessed that employed caregivers (60%) missed 7% ± 12.7% of work hours per week [35].

All data represent costs from 2022 and are shown in TABLE 1, TABLE 2. A discount rate of 3%/y was applied because of the lifetime horizon.

### Health state utilities

Utilities are a measure of preference between health states, where preference can be equated with value or desirability. Utilities for health states included in the model were obtained from international literature and, if necessary, re-expressed on a utility-scale from 0 to 1 (where 0 represents death and 1 represents full health) by using weighting factors. The QALY concept allows combining the effects of health interventions on quantity and the quality of the remaining life years into a single index. QALYs were calculated by multiplying the length of time spent in a certain health state by the utility score associated with it [36].

QoL associated with PS requirement in patients with SBS-IF was extracted from a publication determining utility among 100 patients, which was also used in other health economic evaluations. Health-related QoL was measured with the European Quality of Life 5 Dimensions (EQ-5D) questionnaire [15,37].

To calculate the QoL impact per AE, the disutility per AE was multiplied by the event duration. The treatment-specific incidence of events was then used to calculate the disutility of each treatment. Data on the disutility because of events were derived from different literature sources.

SBS-IF not only affects the patient but also the QoL of carers. To quantify this impact, we followed the approach of the company submission to NICE (Reference number: TA804) and used midpoint utility values of those elicited from a Delphi process and mean EQ-5D values assessed via a carer survey [22].

All utilities were adjusted to the cycle length. Our analysis was based on the assumption that these utilities can be applied to the Austrian population. All utilities are shown in Table 4.

**TABLE 4:** Model inputs: utilities

Utilities per health state		
Health state	Utility/disutility	Source
PS0 d/wk (independent of PS)	0.840	Raghu et al. [15] 2020, Ballinger et al. [37] 2018
PS1 d/wk	0.770	
PS2 d/wk	0.700	
PS3 d/wk	0.630	
PS4 d/wk	0.560	
PS5 d/wk	0.490	
PS6 d/wk	0.420	
PS7 d/wk	0.350	
Disutilities of AE		
Abdominal pain	–0.070	Tielemans et al. [54] 2013
Nausea	–0.100	Tielemans et al. [54] 2013
Gastrointestinal stoma change	–0.072	Average of AE disutilities
Abdominal distension	–0.070	As abdominal pain
Central line systemic infections	–0.070	Worbes-Cerezo et al. [35] 2019
Peripheral edema	–0.072	Average of AE disutilities
Urinary tract infection	–0.070	Worbes-Cerezo et al. [55] 2019
Flatulence	–0.020	Tielemans et al. [54] 2013
Vomiting	–0.150	Tielemans et al. [54] 2013
Fatigue	–0.115	Geale et al. [56] 2017
Pyrexia	–0.010	Geale et al. [56] 2017
Diarrhea	–0.150	Geale et al. [56] 2017
Weight increase	–0.099	Geale et al. [56] 2017
Dyspnea	–0.003	Sullivan et al. [57] 2011
Nasopharyngitis	–0.010	Geale et al. [56] 2017
Caregiver utilities		
PS0 d/wk (independent of PS)	1.00	Scotland et al. [22] 2017
PS1 d/wk	0.95	
PS2 d/wk	0.95	
PS3 d/wk	0.86	
PS4 d/wk	0.72	
PS5 d/wk	0.81	
PS6 d/wk	0.75	
PS7 d/wk	0.74	

Abbreviations: AE, adverse event; PS, parenteral support.

### Sensitivity analysis

A deterministic 1-way sensitivity analysis was done to assess how variations of individual input parameter values affect the model outputs, specifically, the resulting ICUR, and thus to judge the robustness of our findings. Input ranges for sensitivity analysis were obtained from 95% confidence intervals (CIs) when available. Otherwise (e.g., for costs), input ranges were derived by adding or subtracting percentage values to or from the baseline estimates. In addition, a probabilistic sensitivity analysis (PSA) was carried out. This global PSA allows the contribution of each parameter to model outcomes to be investigated while also taking into account the uncertainty of other model parameters. For this purpose, we incorporated a probability distribution of the input variables by means of a second-order Monte Carlo simulation. Each simulation was based on a different value drawn randomly from the distribution of each variable. Second-order Monte Carlo, simulations of 1000 hypothetical patients, were carried out based on the distributions of all input variables: gamma distribution for costs and beta distribution for probabilities and utilities.

## Results

### Cost-effectiveness results

Table 5 presents the model’s average outcomes per patient (costs, QALYs, and LYs) with teduglutide and with no teduglutide. Based on the health state transition probabilities from Harpain et al. [10], teduglutide leads to costs of 2,296,311 € per patient and 10.78 QALYs (13.74 LYs) over a life-time-horizon. No teduglutide is associated with 1,236,816 € and 2.24 QALYs (8.57 LYs). Teduglutide is associated with additional costs of ∼1,059,495 € per patient and a QALY gain of 8.55 or 8 y and 7 mo in perfect health. Caregiver disutilities are lower with teduglutide than in the comparator group and result in a QALY gain of 1.15 or 13.8 mo in perfect health. Patients receiving teduglutide exhibit cost-savings for PS costs plus supplementation (–372,450 €), PS home nursing (–321,379 €), and indirect costs (–140,975 €). Consequently, from the societal perspective, the ICUR amounts to 123,945 €. Taking into account only the healthcare system, the ICUR is 162,225 €. Assuming a WTP threshold of 300,000 € for ultraorphan diseases for each QALY, a (net monetary benefit = WTP ∗Δ QALYs – Δ Costs) of 1,363,955 € was calculated. After 5 y, the average PS days in the teduglutide group are <1, and 98% of patients reach enteral autonomy; in the group without teduglutide, average PS days are 5.55 and 0.2% with enteral autonomy (Supplemental Figure 3).

**TABLE 5:** Cost-effectiveness results (base case)

	Teduglutide	No teduglutide
Teduglutide health state transition probabilities based on Harpain et al. [10] (2021)		
Direct costs		
Costs Revestive PS costs plus supplementation PS home nursing Monitoring costs AE costs	1,992,927.27 € 34,030.68 € 29,364.29 € 47,403.40 € 67,351.22 €	406,480.91 € 350.743.06 € 29,584.92 € 183,797.56.28 €
Total direct costs	2,171,076.22 €	970,606.45 €
Indirect costs		
Employment of patients Caregiver time Caregiver work absenteeism	11,493,848 € 9217.63 € 1077.90 €	143,251.45 € 110,100.38 € 12,857.42 €
Total indirect costs	125,234.02 €	266,209.25 €
Total costs 95% CI	2,296,310.87 € (1,967,148, 2610.770)	1,236,815.71 € (1,146,551, 1,326,628)
Cost difference	1,059,495.16 €	
QALYs		
QALYs patient	10.889	3.489
Caregiver disutilities	–0.105	–1.253
QALYs	10.784	2.235
QALY difference	8.548	
ICUR/QALY(societal perspective)	123,945.35 €	
LYs		
LYs	13.737	8.574
LY difference	5.164	
ICER/LY	232,478.57 €	
ICUR/QALY (health care system)	162,225.01 €	
NMB	1,363,954.52 €	

ICUR: (C _Teduglutid_ − C _Control_) − (QALYs _Tedaglutid_ − QALYs _Control_).

ICER: (C _Teduglutid_ − C _Control_) − (LYs _Teduglutid_ − LYs _Control_).

NMB: WTP∗Δ QALYs − Δ Costs.

AE, adverse event; CI, confidence interval; ICER, incremental cost-effectiveness ratio; ICUR, incremental cost-utility ratio; LY, life year; NMB, net monetary benefit; PS, parenteral support; QALY, quality-adjusted life year; WTP, willingness to pay.

In the case of using transition probabilities for health states derived from the data set of the pooled observational studies, the life expectancy in the teduglutide group amounts to 7.15 QALYs (11.69 LYs). The average PS requirement after 5 y was 1.72 d/wk, and 59.5% of patients reached enteral autonomy. These results in an ICUR of 184,961 €.

**FIGURE 2:**
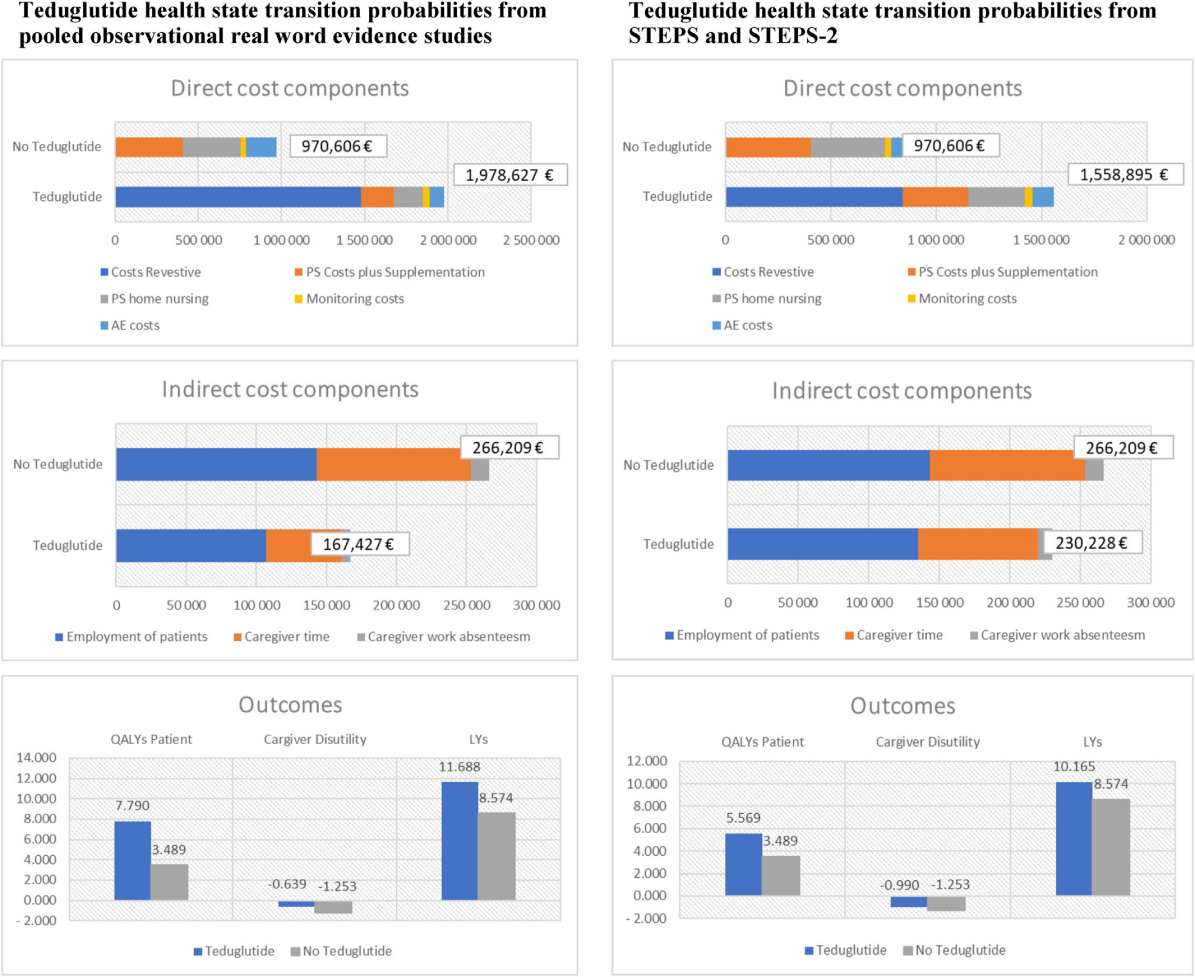
Cost-effectiveness results with different transition probabilities for health states. AE, adverse event; LY, life year; PS, parenteral support; QALY, quality-adjusted life year; STEPS, study of teduglutide effectiveness in parenteral nutrition-dependent short bowel syndrome subjects.

If transition probabilities of health states based on the STEPS studies were used, the total costs in the teduglutide group would be lower because of the reduced life expectancy of 4.58 QALYs (10.17 LYs). The QALY benefit of patients with SBS-IF is reduced to 2.34 QALYs or 28.1 mo in perfect health. The ICUR increased to 235,612 €. The average PS days after 5 y are 3.36 d, and 30.6% of patients reach enteral autonomy. See Figure 2 for the cost-effectiveness results of the pooled observational studies and STEPS studies.

### Deterministic 1-way sensitivity analysis

**FIGURE 3:**
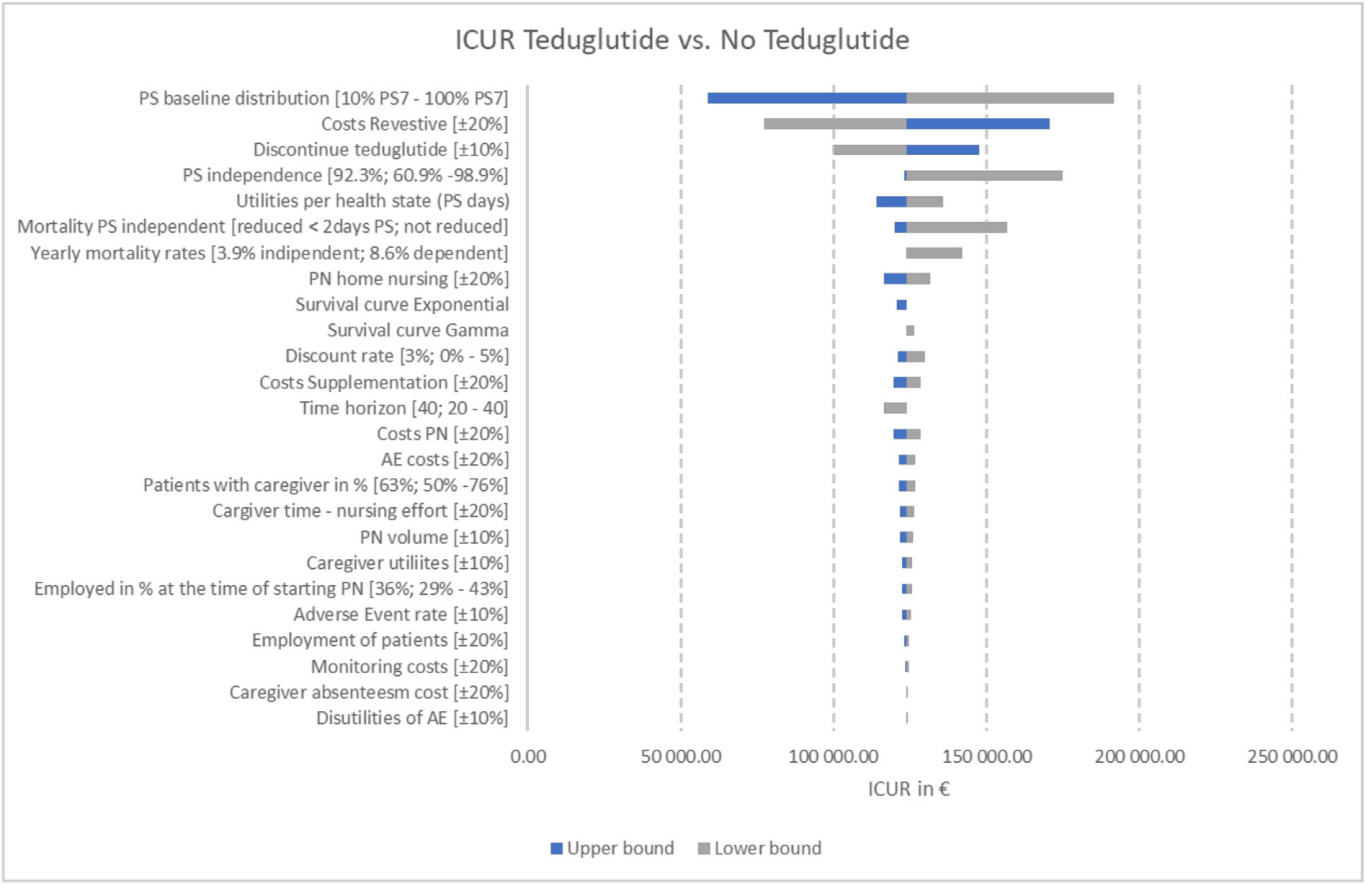
Deterministic sensitivity analysis visualized as Tornado plots where each bar represents a 1-way sensitivity analysis and the length of the bars represents the impact on model results. Deterministic sensitivity analysis was used to identify the critical variables affecting risk analysis. The ICUR per patient is plotted on the x-axis. AE, adverse event; ICUR, incremental cost-utility ratio, PN, parenteral nutrition, PS, parenteral support.

One-way sensitivity analysis (Figure 3) indicated that within the model parameter uncertainty, the area of greatest impact was the PS baseline distribution, costs of teduglutide (Revestive), and the discontinuation rate of teduglutide. Variations in the inputs monitoring costs, caregiver absenteeism costs, and disutilities because of AE have no influence on the result. These observations were congruent when the pooled data or STEPS data set was used. Tornado diagrams for probabilities of health states based on the pooled observational data set and the STEPS data set are presented in Supplemental Figure 4.

### PSA

**FIGURE 4:**
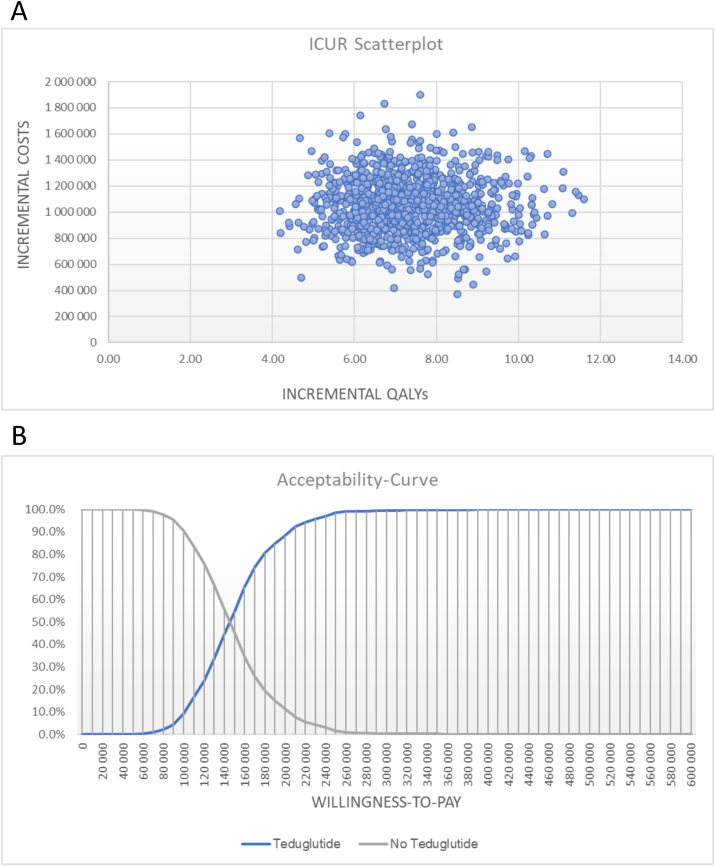
(A) Probabilistic sensitivity analysis. The Scatterplot shows the results of the Monte Carlo probabilistic sensitivity analysis for 1000 patients. Incremental costs (€) are plotted on the y-axis, and incremental effectiveness (QALY) is plotted on the x-axis. The location of the majority of points in the upper right quadrant shows that the simulations yield additional cost and improved health outcomes. The base case results attest to the model’s low level of uncertainty. (B) Cost-effectiveness acceptability curves display the percentage of iterations that favor teduglutide in comparison with no teduglutide over a range of willingness to pay. The x-axis displays reported values as € per QALY. QALY, quality-adjusted life year.

To characterize the full impact of parameter uncertainty, a PSA was undertaken. PSA varies all inputs simultaneously based on their distributional information. The results of the PSA are presented with the help of scatterplots. The points in the diagrams – i.e., all simulations – present the incremental costs and incremental effectiveness (QALYs) of teduglutide compared with no teduglutide. The results of 1000 PSA iterations are presented in Figure 4A.

The mean incremental QALYs gained from teduglutide were 7.35 (95% CI: 6.38, 8.00). The mean incremental costs were 1,051,573 € (95% CI: 739,057, 1,435,994 €). The resulting probabilistic ICUR from 1000 iterations was 143,114 €. The probabilistic results were consistent with the results from the deterministic base case.

The results are also presented graphically in the form of a cost-effectiveness acceptability curve (CEAC). The CEAC plot is a graph that shows the probability of being the most cost-effective therapy at different WTP thresholds. Both interventions, teduglutide and no teduglutide, are plotted on this graph. If a threshold for ultraorphan diseases of 300,000 € is assumed, teduglutide is cost effective in around 99.6% of all simulations. See Figure 4B for the CEAC plot.

If transition probabilities of health states are based on the pooled observational data set, the probabilistic ICUR amounts to 205,731 €, and in the case of a WTP of 300,000 €, 86.5% of simulations are cost effective. In the case of using transition probabilities for health states derived from the STEPS data set, the ICUR is 235,008 €, and 66.7% of simulations are assumed to be cost effective (Supplemental Figure 5).

## Discussion

Here, we demonstrate for the first time that teduglutide therapy meets conventional cost-effectiveness criteria in adult patients with SBS-IF compared with BSC treatment only. A crucial goal in the treatment of patients with SBS-IF with teduglutide is the weaning of PS with the optimal outcome of enteral autonomy. The potential of the intestinotrophic glucagon-like peptide 2 analogue teduglutide to reduce PS dependency has been demonstrated in prospective randomized controlled trials as well as several retrospective observational cohort studies. Nevertheless, clinical effectiveness varies widely throughout the literature: although responder rates in the 2 prospective studies by Jeppesen et al. [17,38] are described to be as high as 63% and 46% after 24 wk, these rates tend to be higher in the published retrospective cohort analyses ranging ≤85%, respectively [20]. Our experience, published by Harpain et al. [10], showed a responder rate of even 100% after 6 mo of treatment. Concerning enteral autonomy rates, these differences are even more blatant [10,17]. Interestingly, by adapting and liberating the strict weaning protocol in the randomized controlled trials, Jeppesen et al. [17,38] were also able to improve the responder rate from 6%–30% in the respective placebo group. We believe that embedding patients with SBS-IF into a multiprofessional team with a tight-meshed, patient-tailored treatment approach, unbound to strict weaning protocols, allows a faster and more substantial reduction of PS. These observations highlight the difficulty of defining the exact efficiency potential of teduglutide in weaning PS in patients with SBS-IF. In order to accommodate these possibilities in our cost-effectiveness analysis, we created 3 different scenarios for the underlying Markov model based on the published literature. The first scenario described our experience with teduglutide to picture the Austrian clinical practice [10]. Second, we pooled PS transition probabilities of 6 observational cohort studies [10,[18], [19], [20], [21],^23^], and the third scenario delineated the pivotal prospective randomized controlled phase III STEPS trial and its open-label extension (STEPS 2) [17,24]. PS dependency significantly affects survival in patients with SBS-IF and, consequently total costs of a life-preserving therapy regimen [29]. In addition, the duration of PS dependency also decreases survival [39]. Unfortunately, to date, precise data on survival in relation to the exact number of PS-dependent days is still missing, and available data on survival refers to an SBS-IF patient collective that may have been outdated by recent scientific-technologic advancements (e.g., the study by Amiot et al. [29] refers to patients enrolled between 1980 and 2006). However, more recent data confirm these results and describe a correlation between PS dependency and survival [40,41]. Nevertheless, assuming a correlation between treatment effect, PS dependency, and survival carries risk of an outcome bias. Because only limited data are available from the clinical STEPS trials, we modeled our mortality rate under consideration of the published 10-y survival data of Amiot et al. [29] using parametric survival curves. It is not possible to distinguish between the reasons why a patient achieves PS independence. PS dependence and independence were considered relevant, as clinical experts had commented during the NICE submission that people with SBS have a near-normal life expectancy once weaned off PS. The incorporated mortality rates are only partly because of central line removal, which would be in line with the assessment of the clinical experts. The cost-effectiveness model of Raghu et al. [15,16] adopts the base case assumption of the mortality rate of the general population in case of PS independence. This would greatly favor teduglutide. Previous observations attributed the high death rate among patients with SBS-IF to a large degree to PS-related AEs [8,42], and studies that evaluated clinical outcome and compared PS-independent to PS-dependent patients with SBS-IF describe comparable cohorts with no significant differences in baseline characteristics [8,29]. In order to extrapolate these survival curves to a lifetime horizon, a Weibull curve was applied based on the results of the Akaike information criterion and Bayesian information criterion. Under these circumstances, mortality is a subject of different sensitivity analyses. Besides the PS weaning probabilities and survival rates, we considered further aspects in our analysis in order to create a precise and realistic cost-effectiveness analysis. First of all, we linked direct medical costs assigned to each health state. Furthermore, medical sequelae of the SBS-IF are another significant factor that needs consideration in a cost-effectiveness analysis. Although PS is a life-saving measure in patients with SBS-IF, one needs to consider that at the same time, PS may be associated with severe complications and, therefore, responsible for the high morbidity and mortality in this patient collective [43,44]. Most complications are related to the central venous catheter and the PS composition [1]. The most common catheter-related complication is CLABSI, causing most of the morbidity and hospital admissions in the SBS-IF patient collective [[44], [45], [46]]. Patients with home PN have described CLABSI rates from 0.38 to 4.58/1000 catheter d [47]. The treatment of these infections generates high outpatient as well as inpatient therapy-associated costs, mounting ≤25,000 € per event [13]. Furthermore, we included costs that resulted from AEs of the teduglutide therapy in accordance with the published adverse event frequencies of the pivotal prospective randomized controlled trial by Jeppesen et al. [17]. In addition, patients with SBS-IF suffer from a significantly reduced health-related QoL [48]. The burden of the SBS-IF highly affects not only the personal but also the professional lives of the affected individuals as well as family members. Activities of daily living, sleeping habits, as well as work capability may be disturbed because of connection times to PS of ≤16 h/ d and increased nocturnal urination because of nightly infusions [49,50]. An altered body image and reduced self-esteem, in combination with disturbed self-perception, may lead to social isolation [51]. Therefore, we included indirect costs, including disease-associated work incapacity, caregiver costs, and work absenteeism of caring family members, in order to include to socioeconomic impact of this burdensome disease in our calculations. With our analysis, we demonstrate that the use of teduglutide in patients with SBS-IF who are dependent on PS is cost effective while significantly improving QoL. An ICUR of 123,945 € was obtained. When the model is analyzed probabilistically, the base case ICER increases to 143,114 €. The key drivers of incremental cost are the PS baseline distribution, teduglutide acquisition costs, and the discontinuation rate of teduglutide. According to the STEPS trial, 52% of patients are distributed in health state PS7 when entering the model [17]. If the percentage is reduced to 10%, the ICUR deteriorates to 191,398 €. If the proportion of patients in state PS7 is 100%, the ICUR improves to 58,828 €. The reason for this is the higher discontinuation rate of teduglutide in stage PS7; the more patients drop out, the lower the incremental costs and thus the ICUR. A change in discontinuation rate of (0% and 5%) results in an ICUR range between 120,967 € and 129,585 €. With regard to the price impact of teduglutide (Revestive), it must be noted that the product is not in the reimbursement code (positive list) in Austria and must be approved individually by a chief physician of the health insurance fund. There is no pricing model in place. The additional costs per QALY gained, or ICUR was taken as the benchmark for a cost-effectiveness threshold. Setting ICER thresholds for drugs for ultraorphan diseases presents unique challenges as the patient population is exceptionally small, making traditional cost-effectiveness calculations difficult. Striking a balance between ensuring access to life-saving treatments for rare diseases and maintaining affordability remains a critical concern in the evaluation of ultraorphan drugs. The “Orphan Medicinal Product Regulation” defines orphan medical products as products for the “diagnosis, prevention or treatment of life-threatening or very serious conditions that affect no more than 5 in 10,000 people in the European Union” [52]. NICE, for example, evaluates medicines through a technology assessment process. A small subset of medicines for very rare conditions meets the strict criteria to follow the highly specialized technology evaluation process with an ICER threshold higher than £100,000 (114,077 €) per QALY gained. To meet the requirements, the technology must be licensed for a chronic and severely disabling condition, target a small patient population, and be used within a highly specialized service in very few national health service centers [53]. Teduglutide was rejected from the highly specialized technology program; nevertheless, the appraisals for teduglutide resulted in a positive recommendation following an appraisal consultation meeting [14]. Only 2 cost-effectiveness studies have been conducted to assess the value of teduglutide in adult patients. Raghu et al. [15] evaluated the cost-effectiveness of using teduglutide in United States adult patients with SBS. Results revealed costs of $949,910 (886,551 €) per QALY gained. Teduglutide therapy was cost-saving in 13% of model iterations. The second analysis was the teduglutide company submission from Takeda. Both calculations lack a societal perspective, only calculating with direct medical costs and thereby may miss out on the true financial impact of teduglutide therapy. Furthermore, both studies only used the PS weaning probabilities of the STEPS study, thereby potentially missing out on the “real” therapeutic potential of teduglutide. The key strength of the present model is that it faced the uncertainty in teduglutide effectiveness by creating 3 different scenarios regarding the weaning potential of teduglutide, thereby covering the published literature on teduglutide effectiveness and improving the generalizability of the results. In addition, indirect costs were included in the calculations to quantify the total impact of costs and consequences. When interpreting the results of this publication, the limitations of a Markov model need to be considered. A Markov model assumes that a future state of the system is dependent only on the current state and not on the sequence of events that led to the current state. Interacting variables and nonlinear relationships cannot be considered, and thereby, the model may have missed out on the complexity of the disease setting. Furthermore, this stochastic model assumes that the population has a predetermined probability (e.g., PS weaning probability) of experiencing an outcome. Applying 1 single measure to this complex patient population limits the ability to identify a subpopulation that may experience a greater clinical benefit and subsequently is more cost effective with the teduglutide therapy. There is a large heterogeneity that exists in the SBS-IF population, most likely because of differences in the remnant bowel anatomy, interindividual differences in spontaneous intestinal adaptation, and different treatment approaches. The trials that have evaluated teduglutide to prove its efficacy show a large degree of uncertainty, which is likely reflective of that heterogeneity. Therefore, we created 3 different “scenarios” regarding the PS weaning ability on the basis of the available literature in order to cover a broad range of possible probabilities. Moreover, a head-to-head comparison was not available in the case of observational studies. Finally, parameter uncertainty was minimized by conducting deterministic, 1-way, and probabilistic sensitivity analyses.

In summary, teduglutide is cost effective in the treatment of adult patients with SBS-IF from a societal perspective. Our results highlight the importance of early and high enteral autonomy rates and their significance on the socioeconomic outcome. Future research needs to address the issue of SBS-IF patient heterogeneity and thereby enable patient identification of those who are most likely to reach enteral autonomy.

## Author contributions

The authors’ responsibilities were as follows – EW, FH: conception, design, acquisition of data, analysis and interpretation of data, and drafting of the article; CD, AS: conception, design, acquisition of data, and critical revision of the article; EH: acquisition of data and critical revision of the article and all authors: read and approved the final manuscript.

## Funding

The authors reported no funding received for this study.

## Data availability

Data will be made available on reasonable request.

## Conflict of interest

EH and AS report lecture fees from Takeda outside the submitted work. FH reports a grant and lecture fee from Takeda outside the submitted work. All other authors report no conflicts of interest.
