# Supplementary material for: Cost-effectiveness of teduglutide in adult patients with short bowel syndrome – a European socioeconomic perspective
Source: Am J Clin Nutr. 2024 Mar 1;119(5):1187–99. doi: 10.1016/j.ajcnut.2024.02.031 (PMC11130673; doi:10.1016/j.ajcnut.2024.02.031)
Supplement: Multimedia component 1 [file mmc1.docx]

SUPPLEMENTARY MATERIAL

**Cost-effectiveness of teduglutide in adult patients with short bowel syndrome – a European socioeconomic perspective.**

Evelyn Walter

**SUPPLEMENTARY APPENDIX**

**Figure S1: Pooled PS independence rate from the six observational studies**


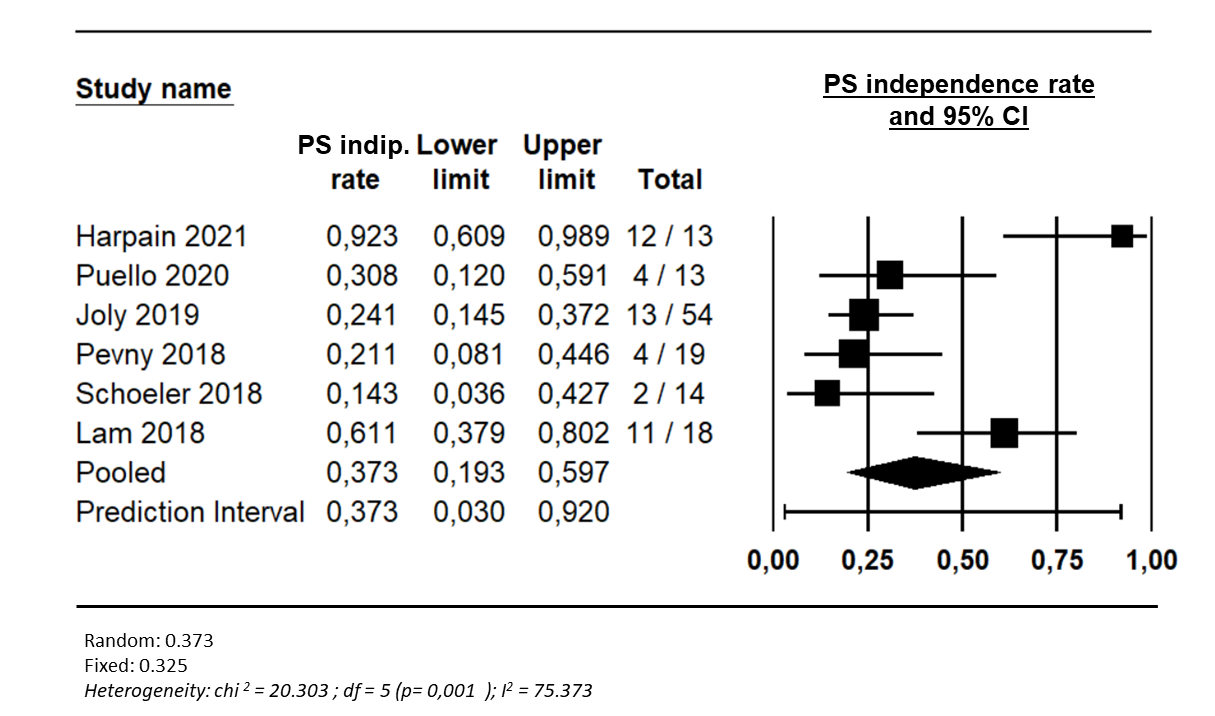


**Figure S2. Survival curves.**


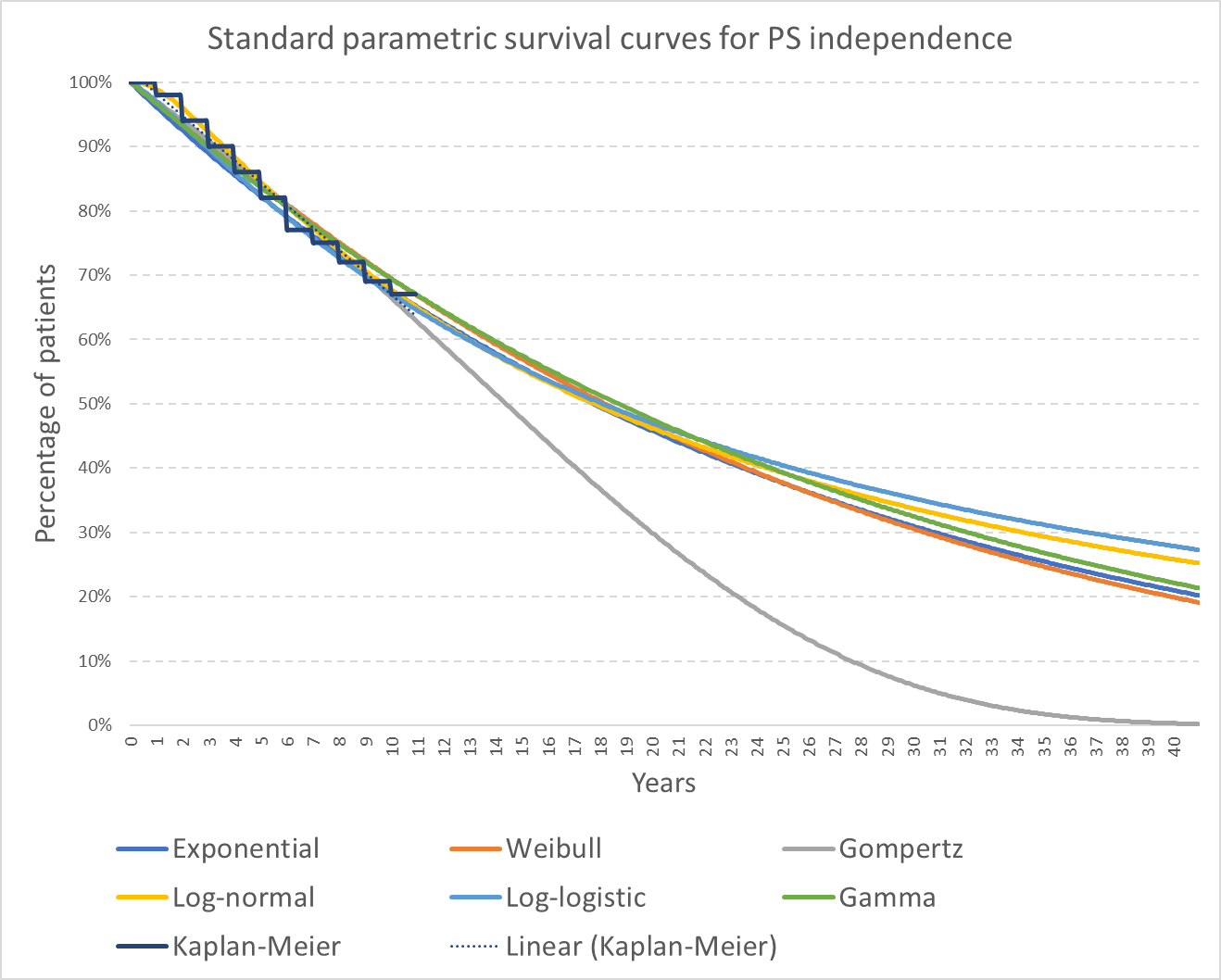

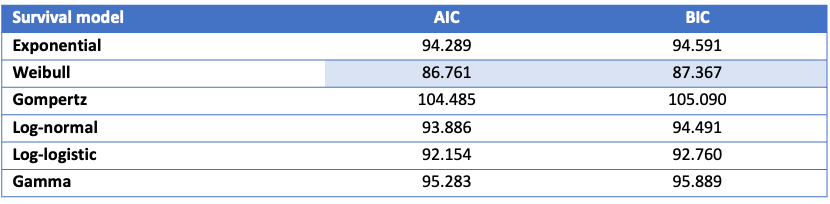


**
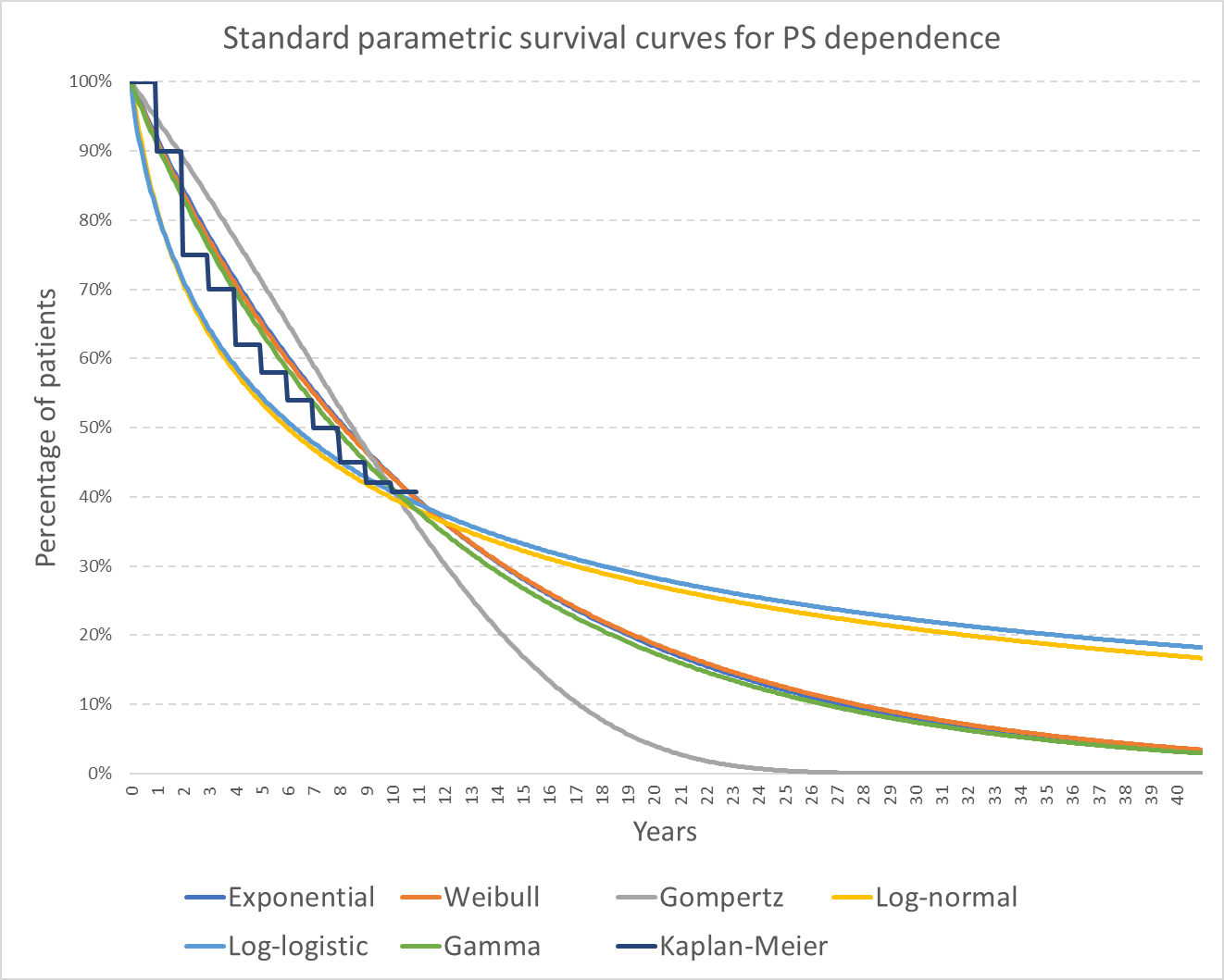

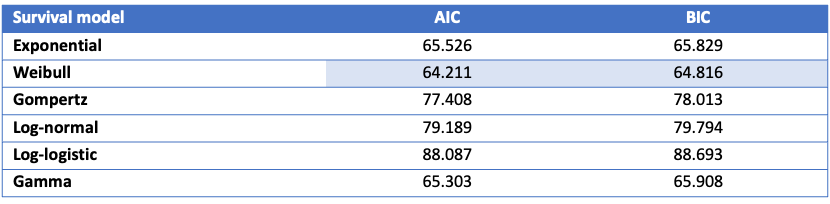
**

**Figure S3. Distribution of health states** in percentage [%] (Y-axis) over time [years] (Y-axis).

**Teduglutide health state transition probabilities based on Harpain et al. 2022**

**
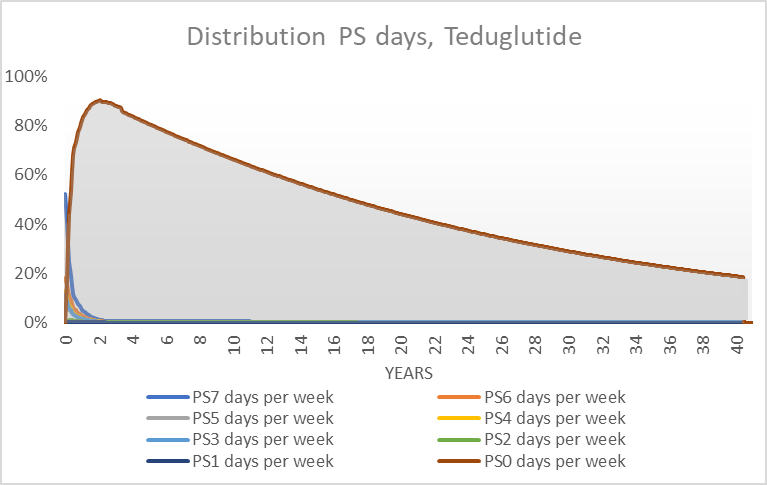

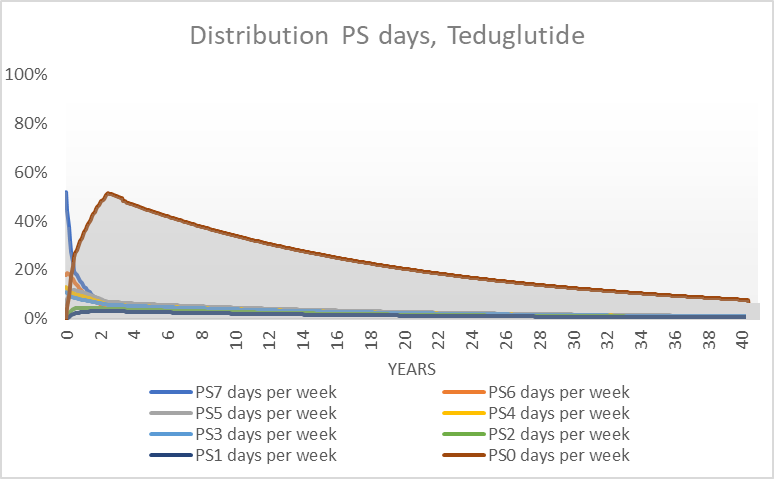

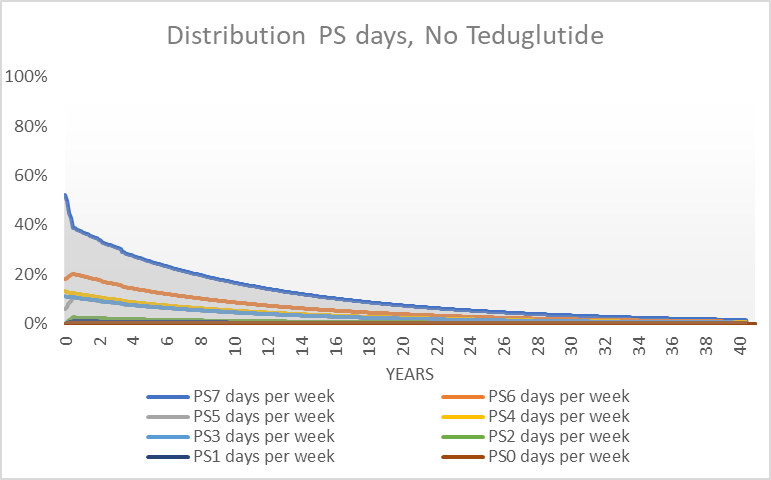

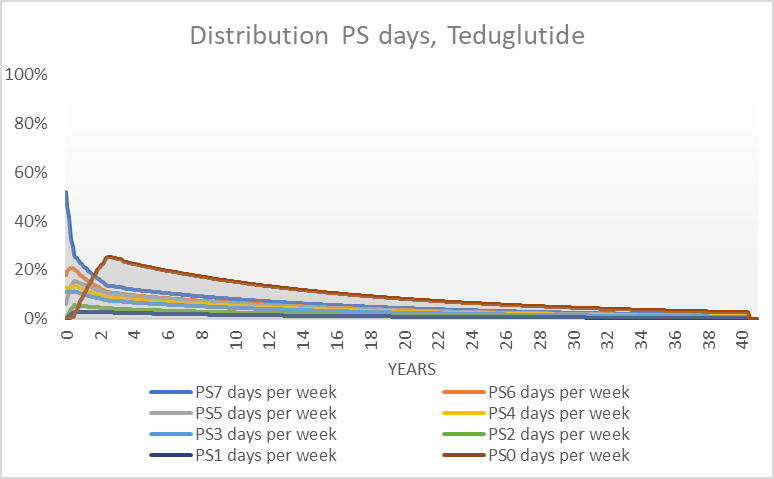
**

**Teduglutide health state transition probabilities from pooled observational real word evidence studies**

**Teduglutide health state transition probabilities from STEPS and STEPS2**

**Figure S4. Deterministic Sensitivity Analysis.**

**
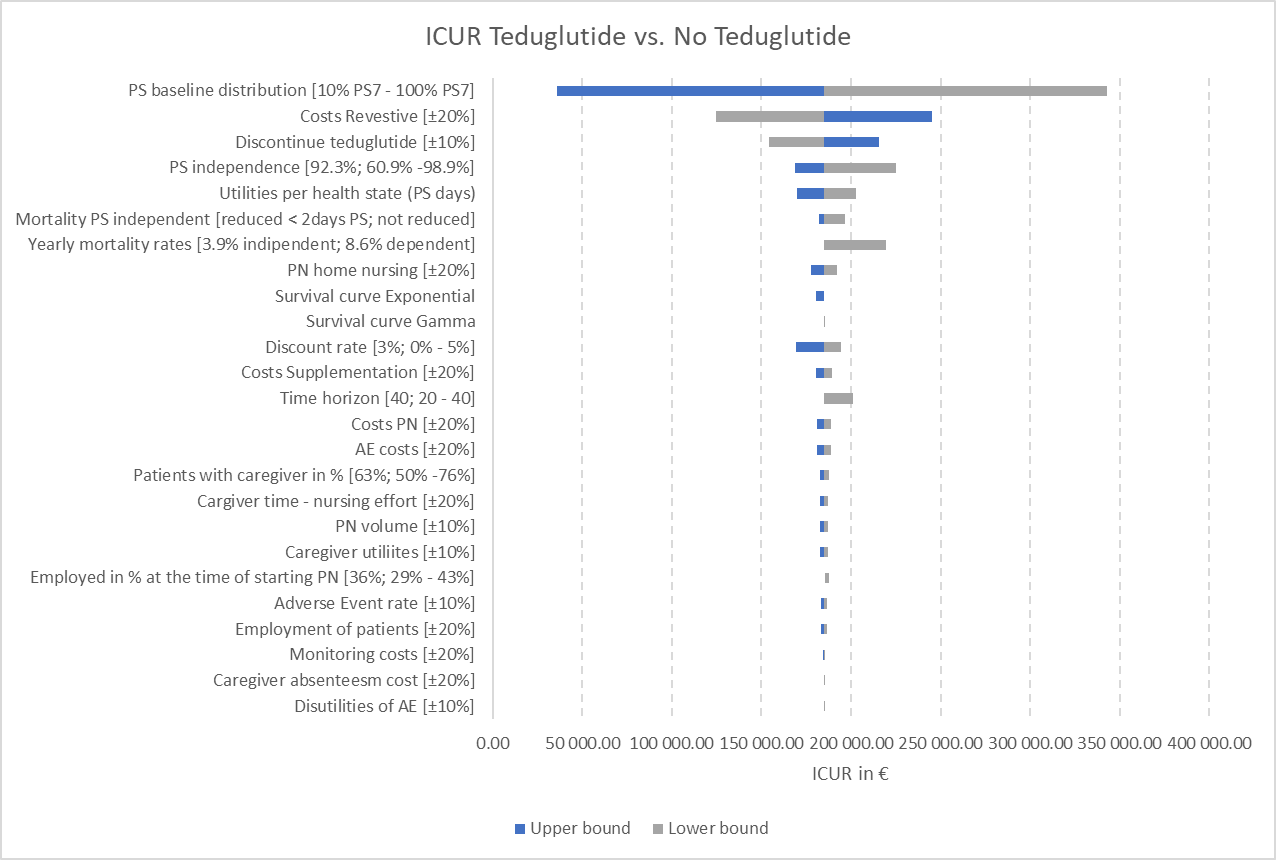

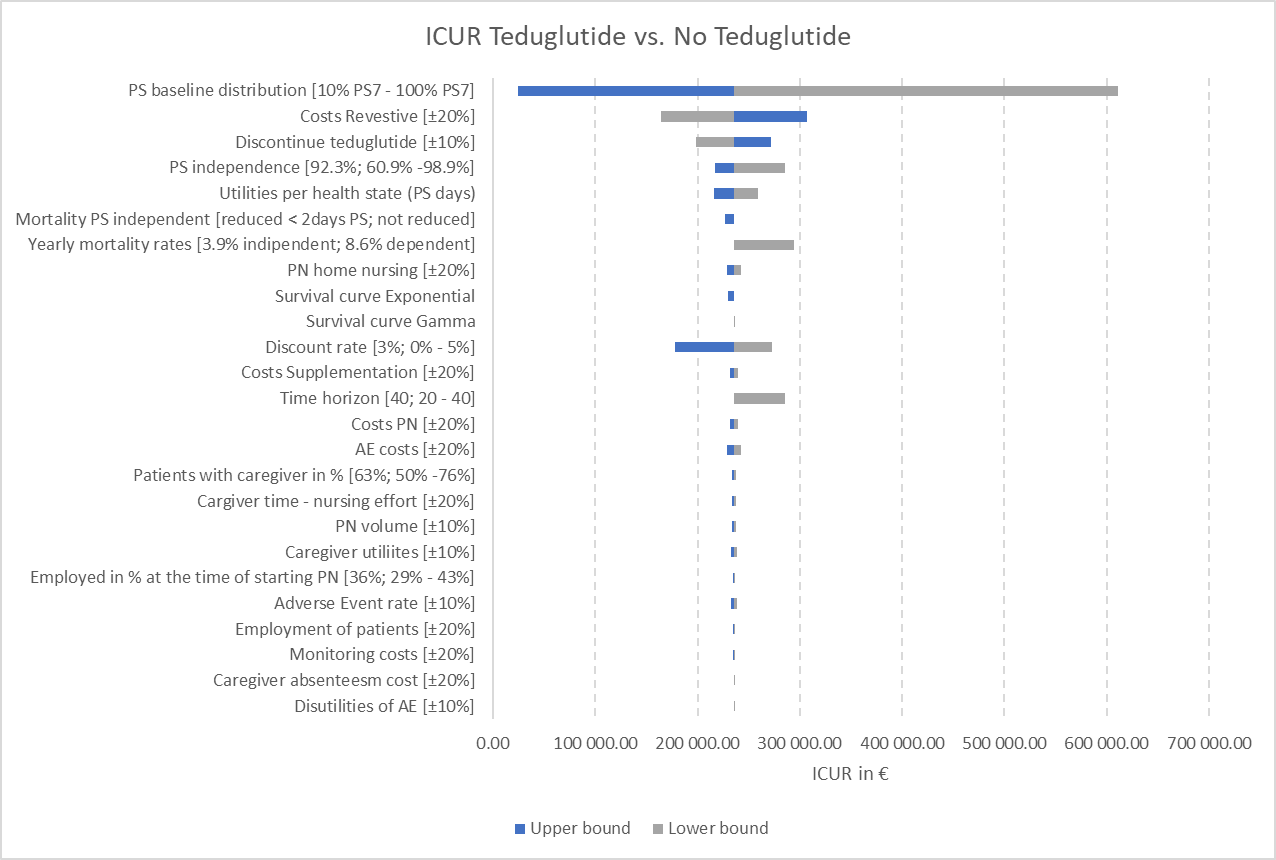
**

**Teduglutide health state transition probabilities from pooled observational real word evidence studies**

**Teduglutide health state transition probabilities from STEPS and STEPS-2**

**Figure S5.** **Probabilistic Sensitivity Analysis** with different transition probabilities for health states.


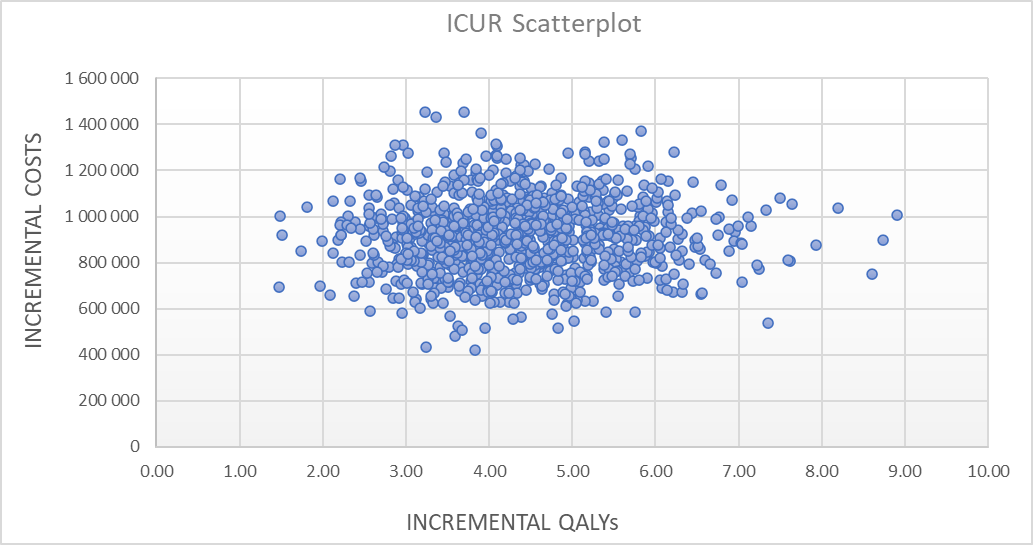

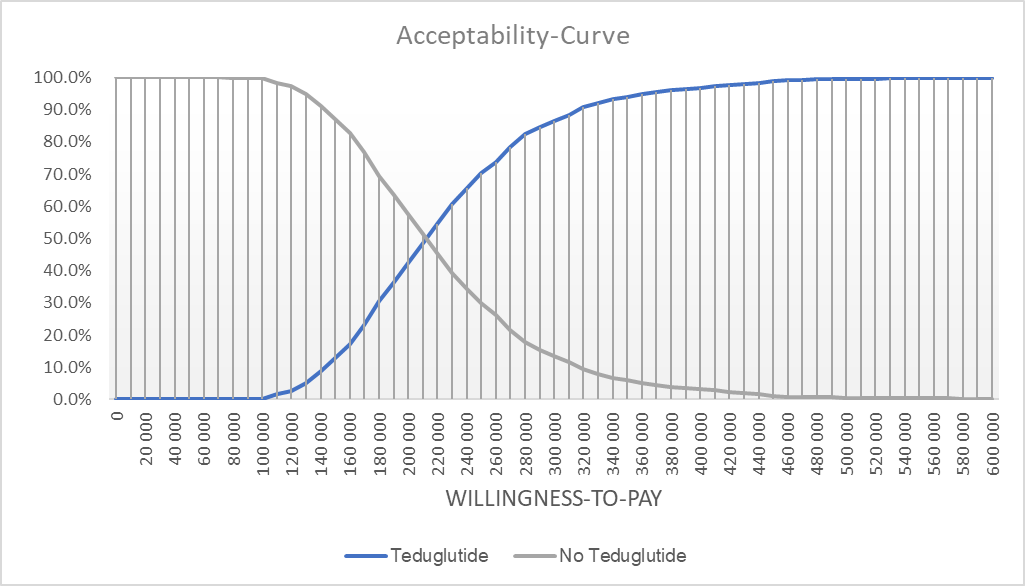

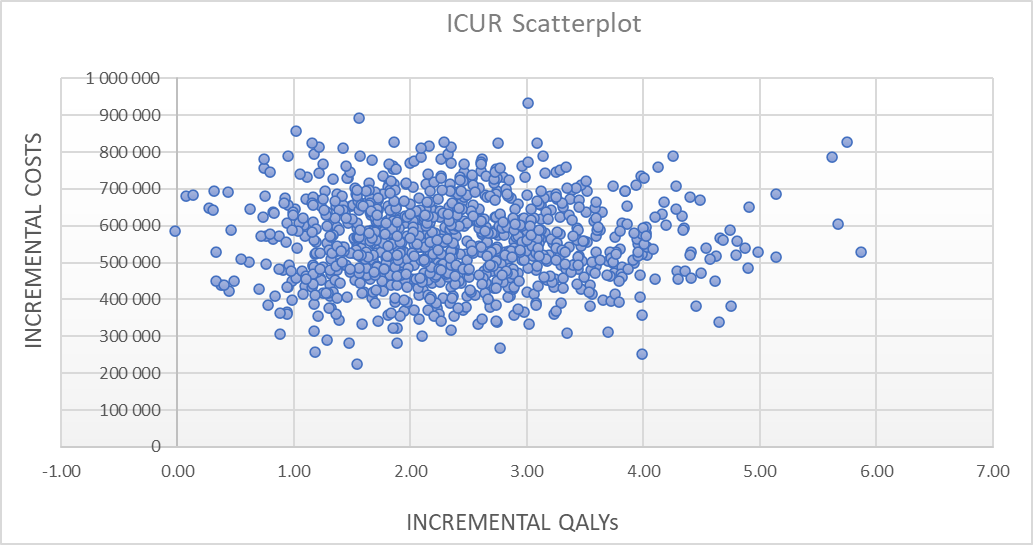

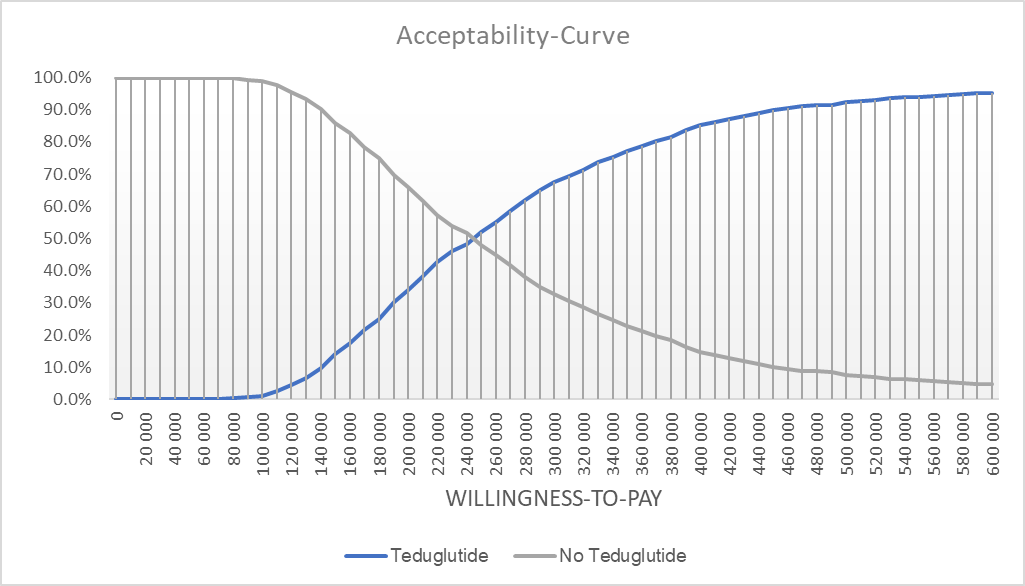


**Teduglutide health state transition probabilities from pooled observational real word evidence studies**

**Teduglutide health state transition probabilities from STEPS and STEPS-2**

| Table S1. Overview of included cohort studies. | | | | | | |
| --- | --- | --- | --- | --- | --- | --- |
| Author | Location | Study type | Median follow-up | Data collection dates  (index to cut-off) | Population | Patients on teduglutide |
| *Harpain et al. 2021 [10]* | Austria | single-center | 107 weeks | 2016 – 2020 | Adult SBS-IF | 13 |
| *Puello et al. 2020 [19]* | USA | single-center | 3.2 years | 2013 – 2019 | Adult SBS-IF | 18 |
| *Joly et al. 2019 [20]* | France | multi-center | 24 weeks | 2015 – 2017 | Adult SBS-IF | 54 |
| *Pevny et al. 2018 [21]* | Germany | single-center | 56 weeks | 2014 – 2017 | Adult SBS-IF | 19 |
| *Schoeler et al. 2018 [23]* | Germany | single-center | 14.5 months | 2014 – 2016 | Adult SBS-IF | 14 |
| *Lam et al. 2018 [18]* | USA | single-center | unknown | 2009 – 2015 | Adult SBS-IF | 18 |

**Figure S1. Pooled enteral autonomy rate from the six observational studies.**

**Figure S2.** **Survival curves.**

AIC *Akaike information criterion*

BIC *Bayesian information criterion*

**Figure S3.** **Distribution of health states** in percentage [%] (Y-axis) over time [years] (X-axis).

PS *Parenteral support*

STEPS *Study of Teduglutide Effectiveness in Parenteral nutrition-dependent Short-bowel syndrome subjects*

**Figure S4. Deterministic Sensitivity Analysis.**

AE *Adverse event*

ICUR *Incremental Cost-Utility Ratio*

PN *Parenteral nutrition*

PS *Parenteral support*

STEPS *Study of Teduglutide Effectiveness in Parenteral nutrition-dependent Short-bowel syndrome subjects*

**Figure S5. Probabilistic Sensitivity Analysis** with different transition probabilities for health states.

STEPS *Study of teduglutide effectiveness in parenteral nutrition-dependent short-bowel syndrome subjects*

ICER *Incremental Cost-Effectiveness Ratio*

QALY *Quality-Adjusted Life Year*

**Table S1.** **Overview of included cohort studies.**
